# Designing a Patient-Friendly Website for Newly Diagnosed Cancer Patients with the Participatory Health Research Approach

**DOI:** 10.3390/ijerph19041969

**Published:** 2022-02-10

**Authors:** Juliane Rackerseder, Carolin Hornbach, Peter Dicks, Hedy Kerek-Bodden, Theresia Krieger

**Affiliations:** 1 Institute of Medical Sociology, Health Services Research, and Rehabilitation Science (IMVR), Faculty of Human Sciences and Faculty of Medicine, University of Cologne, Eupener Str. 129, 50933 Cologne, Germany; carolin.hornbach@uk-koeln.de (C.H.); theresia.krieger@uk-koeln.de (T.K.); 2 Vocational School University Hospital Aix-la-Chapelle, Pauwelstr. 30, 52074 Aix-la-Chapelle, Germany; pdicks@ukaachen.de; 3 District Association of Larynx Operated Aachen e.V., Lörschpülgen 24, 52134 Herzogenrath, Germany; 4 House of the Cancer Patient Support Associations of Germany (HKSH-BV), Thomas-Mann-Str. 40, 53111 Bonn, Germany; kerek-bodden@hausderkrebsselbsthilfe.de

**Keywords:** cancer, patient information material, patient-friendly website, website design, participatory health research, quality assessment, piloting, patient engagement

## Abstract

High-quality and user-friendly patient information material (PIM) is essential for understanding and accepting a new care programme. When optimising the PIM of the integrated, cross-sectoral psycho-oncological (isPO) care programme, the design of the fifth element of the patient information strategy—the patient-friendly website—was still pending. In this paper, the iterative design process of the patient-friendly isPO website is described. We applied the participatory health research (PHR) approach to enable high levels of participation of its respective end-users (e.g., cancer survivors), service providers, and experts. The design included six steps: (1) initiation, (2) planning, (3) initial idea exploration, (4) creation of a first working version, (5) three optimisation loops, and (6) dissemination. An exploratory mixed-methods design has been used. Qualitative data collection included document analysis, interviews, and participatory action research (PAR) loops with focus groups. Finally, the quality of the newly designed website was quantitatively assessed with the UPIM-Check, a user-friendly instrument for assessing and optimising PIM. The PHR approach was indispensable for the design of our needs-oriented, patient-friendly website. Participants’ high levels of participation strongly contributed to the products’ quality. The final descriptive statistical evaluation shows that the final website was rated very good on average by its end-users.

## 1. Introduction

### 1.1. Background Patient Information Material (PIM)

Patient information materials (PIM) play an essential role in improving the patient’s ability to make health-related decisions [1]. PIM (e.g., leaflets, posters, or information packages) are considered to be an integral aspect of promoting the health of patients and aim to support the patient’s health literacy [2,3]. Nowadays, a majority of patients are searching for health information on the internet [4]. Programme webpages are considered to be a powerful tool for informing potential patient participants, e.g., about the recruitment of a new study [5]. In addition, programme websites may improve patients’ understanding and satisfaction with a new programme [6,7]. 

In order to explore PIM’s quality, several criteria and instruments are available. Criteria include both content and formal aspects, consisting of the correctness of content, readability, comprehensibility, and usability [8]. PIM assessment instruments, such as DISCERN or PEMAT (Patient Education Materials Assessment Tool), are instruments that include evaluation scales [9,10]. HON (Health On the Net foundation) is acknowledged as a quality seal, defining the code of conduct for PIM producers [11]. Recently, a new PIM quality assessment instrument, the UPIM-Check (User-Friendly Patient Information Material Checklist), was developed by applying the participatory health research (PHR) approach and demonstrated its effectiveness [12]. The UPIM-Check helps to assess a PIM´s quality in four areas by using a traffic light system [12]. In addition, improvement suggestions can be noted in an adjacent text field. Due to the “intuitive to use” traffic light system, the UPIM-Check proved to be suitable for end-users’ (e.g., cancer patients) and experts’ PIM quality assessments [13].

### 1.2. The Need for a Patient-Friendly Website for a New Psycho-Oncological Care Programme

The majority of adults utilize the internet for health information [14,15,16]. The importance of digital PIM within healthcare interventions has increased within the past few years, as they demonstrated a means to improve access to essential healthcare information [17,18,19,20]. In Germany, a new integrated, cross-sectoral psycho-oncological care programme (isPO) was designed, implemented, and evaluated between 2017 and 2022 [21]. Its overall goal is to implement this approach into Germany’s standardised care [21]. isPO strives to establish a new psychosocial and psychotherapeutic stepped-care programme that aims to reduce psychosocial distress among adult cancer patients [22]. To enhance the understandability of the complex programme and the recruitment of the patients, high-quality PIM turned out to be crucial. 

In isPO’s early implementation phase, service providers and patients reported that the initially developed PIM were linguistically inappropriate, too exhaustive, and partly redundant [23]. The isPO project was represented on the Centre for Integrated Care of the University Hospital of Cologne (CIO) website. However, the information provided was useful for other researchers or funders, but not preliminary for patients. These shortcomings led to poor programme acceptance and low participant recruitment. Hence, the optimisation of the isPO PIM was needed, and as such was conducted between October 2019–February 2020. It resulted in four high-quality PIM, namely, a poster, leaflet, patient folder, and one-pager, that were introduced in practice in 03/2020 [12,24]. The PHR approach [25] was applied for the PIM optimisation process, whereby three groups contributed as co-researchers: end-users (former cancer patients), health professionals, and service providers. During the optimisation process, the need for a fifth element—a patient-friendly website—was identified. However, due to resource bottlenecks, this element was separately developed within a Master’s qualification (first author JR) and is the focus of this paper. 

### 1.3. Participatory Approaches in PIM Development Processes

Participatory approaches have a dual objective: (1) the linking of research and intervention and (2) the cooperation at eye level between researchers and participants from the target group [26]. PHR aims to understand social realities and improve society from the bottom up [25,26]. It is characterised by the researchers’ reflective and open-minded attitude and an open process-oriented outcome [27]. 

**Figure 1:**
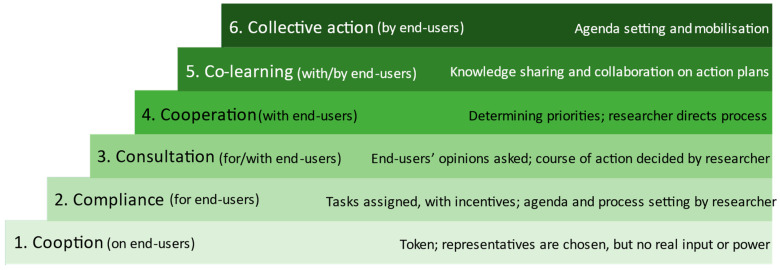
The six participation levels of the participatory health research approach (own figure, adapted from Cornwall, 1996).

Various participation models exist that distinguish between different degrees of participation, e.g., Arnstein’s ladder of participation [28] or Pretty’s typology of participation [29]. Cornwall [30] differentiates six participation levels (Figure 1) that describe the relationship of the target group with the professional researcher and the characteristics of participation during the participatory research process [30].

### 1.4. Objectives and Research Questions of this Study

As a patient-friendly website was identified as the missing element to complete the isPO PIM strategy, we decided to design it with the PHR approach, as this was successfully used during the PIM optimisation process [24]. In this paper, we will describe (1) how the patient-friendly website for isPO was developed and (2) how the final version of this website was evaluated by its end-users (new cancer patients). 

## 2. Materials and Methods

### 2.1. Study Design, Approach, and Participants

For this research project, a QUAL-quant sequential mixed-methods design was chosen [31]. The qualitative part included interviews and focus groups, whereas the quantitative research part consisted of a plausibility check that was conducted with the UPIM-Check [12]. The PHR approach was applied during the whole process. 

By applying PHR, new knowledge was generated from the participation of four groups: (1) potential end-users, represented by members of various cancer self-help groups, (2) isPO-Oncoguides (i.e., former cancer patients), (3) isPO-Case Managers (i.e., the personal contact person for the participant of the isPO project), and (4) experts, being members of the Cancer Society North Rhine-Westphalia (KG NRW). The PHR research team included the primary researchers (JR, TK) who were the impulse providers for this study. Furthermore, the other authors included co-researchers (PD, HKB) and a critical friend (CH). 

In Table 1, the characteristics of the study participants are described. 

The isPO evaluation team, affiliated with the Institute for Medical Sociology, Health Services Research and Rehabilitation Science (IMVR) at the University of Cologne, facilitated the design process by applying the critical friend approach [32].

### 2.2. Research Process

**Figure 2:**
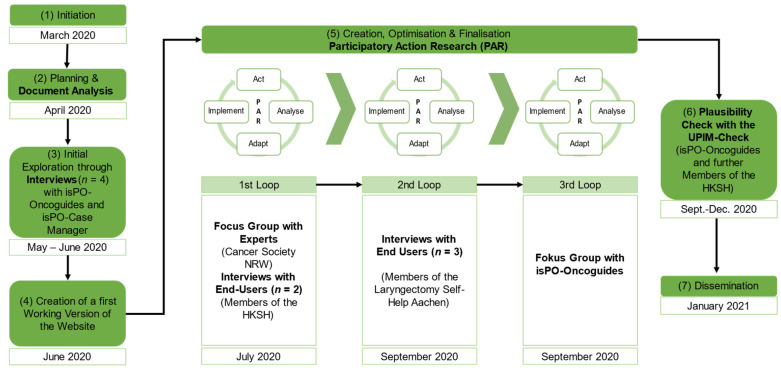
The research process of the website design.

The research process was divided into seven steps: (1) initiation, (2) the planning and document analysis, (3) the first exploration through interviews, (4) the creation of a first version of the website, (5) three optimisation loops and, finally, (6) plausibility check and (7) dissemination (Figure 2).

Participatory action research (PAR) was conducted in step 5 during the three optimisation loops, containing Act, Analyse, Adapt, and Implement [33].

In the following, each research step will be explained: 

***Step 1—Initiation***

In addition to the four previously designed analogue PIM, the missing fifth element—a patient-friendly website—was desired for isPO. Its development process was initiated in March 2020 (Figure 2, left side), and the first contacts with possible peer support organisations were established. The first participation level (instrumentalization) was reached by reading and talking about our target population (Figure 1).

***Step 2—Research process planning and document analysis***

Our planning included: (1) the installation and setup of the WordPress programme [34], (2) setting the administrative conditions for the research steps, and (3) exploring recruitment strategies for PAR participants (e.g., former cancer patients). 

The previously optimised PIM [24] were assessed using the document analysis method. They were chosen as starting material for the data collection, as they were already optimised using the PHR approach, and its informative value and wording was approved to be patient-friendly [12].

***Step 3—Initial exploration through interviews***

To obtain a first impression of patients’ actual needs and requirements for an isPO programme website, four interviews have been conducted (isPO-Oncoguides *n* = 3; isPO-Case Manager *n* = 1) (see Table 1). The isPO-Oncoguides are specially trained to support patients with information communication within isPO. These participants were invited through a personal letter. The fourth interview partner, an isPO-Case manager, was invited and recruited via a direct working contact within the isPO programme. Due to the COVID-19 pandemic, telephone interviews were conducted. In this third step, we reached participation level four (cooperation) with the end-users (Figure 1). 

***Step 4—Creation of an initial website working version***

Based on the condensed knowledge from the document analysis (step 2) and the interviews (step 3), the first version of the patient-friendly website was designed. This process was guided by the criteria of Krug and Dubau [35] that were linked to the merging elements of the interview analyses. Because in this step the website was technically designed by the primary researcher, no active engagement with the target group was performed.

***Step 5—Creation, optimisation, and finalisation***

Our website creation process contains three PAR optimisation loops (Figure 2, middle side). Firstly, data were generated through interviews or focus groups (Act) and assessed (Analyse). Next, the structure and content of the website were improved (Adapt). Finally, optimisations were transferred on the website (Implement). After completing each loop, the current status of the website was critically reflected within the isPO evaluation team in a systematic manner, using a critical-friendly approach [32] to assure its content correctness. 

*Step 5.1 Loop 1*

In this loop, one focus group with three experts from the KG-NRW and two interviews with members of the House of Cancer Self-Help (HKSH) were conducted. The recruitment of the focus group participants was conducted via direct contact by e-mail. Besides the experts of the KG-NRW, one expert from the IMVR evaluation team also participated in the focus group as a critical friend. Due to the COVID-19 pandemic, interviews were held via Zoom video calls. 

A complete PAR loop was conducted. Therefore, in both data collection rounds (focus group and interviews), the screen was split to show the first version of the website to the participants (Act). Next, the website was critically discussed by the participants (Analyse). Third, optimisation suggestions were realised (Adapt) (e.g., structural, textual, or graphical shortcomings). Finally, the results of the analysis were technically incorporated (Implement) into the, at that point, existing version of the website. During our interactive focus group discussion, participation level five (co-creative learning) and during the interviews, participation level four (cooperation) was achieved (Figure 1). 

*Step 5.2 Loop 2*

In loop two, the recruitment of the three interviewees of the District Association of Larynx Operated People Aachen e.V. was conducted by telephone in combination with a personal information letter sent via e-mail. Two of the three interviews were conducted face to face. Interview three was held via telephone. The website was critically observed and reflected upon together with the primary researcher as it was shown on a screen during the two face-to-face interviews, and the PAR steps *Act* and *Analyse* were the same as in loop one. For the telephone interview, the participant received a website link in advance and visited it before the interview. The participant’s statements were, thus, only based on his memory, as he was viewing and analysing the website before the interview. The PAR steps *Adapt* and *Implement* were similar to loop one. We reached the fourth level of participation (cooperation) by engaging the cancer peer groups in the process (Figure 1).

*Step 5.3 Loop 3*

The third and final optimisation loop included a focus group (PAR Act and Analyse). No active recruitment of participants for the focus group was necessary, as it was interlinked with another internal project event of isPO. Seven isPO-Oncoguides participated. Two experts from the IMVR evaluation team stimulated the conversation with questions (critical friend approach). Textual optimisation was realised in parallel to the focus group, and technical issues were solved afterwards (PAR Adapt and Implement). During this final interactive focus group discussion, participation level five (co-creative learning) was achieved (Figure 1). 

***Step 6—Plausibility check***

Subsequently, facilitated by the HKSH-BV support, several members from different peer groups and the participants of the focus group were invited via e-mail to conduct the plausibility check of the new website. For this purpose, the website was open for evaluation for a total of six days. The assessment instrument UPIM-Check was sent to the participants for this purpose, and it included four quality criteria: Q1 correctness and validity of the content (9 items), Q2 readability of content (8 items), Q3 structural readability (4 items), and Q4 graphical readability (10 items) [12]. A comprehensive overview of the UPIM-Check’s quality criteria can be found at https://www.imvr.de/wp-content/uploads/UPIM-Check_English.pdf (accessed on 24 January 2022) [36]. 

Due to the poor response rate, further members of the HKSH-BV were contacted with a personalised cover letter, and the call for completing was prolonged for another 12 days. The checkboxes of the completed UPIM-Checks were evaluated with the help of descriptive statistics using SPSS. This enabled the researcher to gain an overview of the evaluation. In addition, the information in the open text fields was qualitatively assessed (thematic analysis) [37]. Since the practical opinion of the end-users was requested, participation level three (consultation) was achieved (Figure 1). 

***Step 7—Dissemination***

The final, mature version of the patient-friendly website was presented to the isPO project management within a video telephone and split-screen session lasting for two hours (13.11.2020) (Figure 2, right side). Afterwards, the product was submitted to the Joint Federal Committee (G-BA) in the Public Relations and Communication department (funder of isPO) to obtain permission for incorporating the G-BA logo. It was approved for publication by the German Aerospace Centre (DLR) and the G-BA (11.01.2021). Finally, all four isPO care networks interconnected their websites with the newly designed patient-friendly website. During this last step of publishing the website, participation level two (compliance) was achieved (Figure 1).

### 2.3. Data Collection and Analysis

#### 2.3.1. Qualitative Data Collection and Analysis

With the document analysis method [38], the previously optimised four isPO PIM were explored [23]. This process was performed in four steps: (1) the formulation of a research question, (2) the determination of the source material, (3) assessing the significance of the documents, and (4) interpreting the documents. Since the existing PIM are the basis for the patient-friendly website, the following research question was formulated: *which content and graphic elements of the optimised PIM should be used for the website?*

For all interviews and focus groups, a semi-structured guideline was developed and utilised, including the following themes: (1) information on psycho-oncological care, (2) information about the isPO project, (3) the existing PIM, and (4) the construction and design of a patient-friendly website. Before data collection, all participants signed an informed consent form. All sessions were documented with an audio recorder; recordings were transcribed verbatim. Finally, the material was analysed with the thematic analysis method [37], incorporating six steps: (1) becoming familiar with the data, (2) generating initial codes, (3) creating initial themes, (4) reviewing themes, (5) defining themes, and (6) writing the results.

#### 2.3.2. Quantitative Analysis Using the UPIM-Check Instrument for Descriptive Analysis

The plausibility check of the website with the UPIM-Check instrument (Table S1) took place in the third optimisation loop (Figure 2, middle). For assessing the plausibility of the patient-friendly website by its end-users with the UPIM-Check instrument, the following hypothesis was formulated:

**Hypothesis** **1:** For the evaluation of the patient-friendly website, conducted by the target group (cancer patients) with the UPIM-Check, the four quality criteria will be rated very good at an average of at least 50%.

The assessment of the plausibility check was conducted through descriptive analysis. A “measures of frequency” analysis was performed to demonstrate the findings of the UPIM-Check. The findings illuminate the participants’ perception of the strengths and weaknesses of the user-friendly website. 

## 3. Results

Our patient-friendly website for the isPO programme was designed in a participative and iterative process that lasted 11 months (March 2020 to January 2021). It incorporates the main page, four topic pages, one FAQ page and ten sub-pages (https://ispo.uni-koeln.de (accessed on 24 January 2022)). In addition, links to the isPO care networks, figures, and interactive tools aim to facilitate the understandability of the complex stepped psycho-oncological care of isPO, with its different components and its affiliated isPO study. 

In total, 30 people contributed their valuable knowledge and experiences to this process. It is based on approximately 11 h of interviews and 3 h of focus groups. As depicted in Figure 2, new knowledge was generated only during steps two, three, four, five, and six of this study. Outcomes will be presented sequentially in the following. 

### 3.1. Outcomes from Step 2—Document Analysis

As illustrated in Table 2, the four previously optimised isPO PIM [23] were analysed under the aspects of use, content, and design features. The last column shows “what was perceived as most suitable” for a website by the primary researcher.

The four isPO PIM that were already implemented in the care networks demonstrated different highly appreciated aspects that were identified as helpful for the development of this patient-friendly website. The poster and the leaflet (i.e., the green colour scheme and the tree motif) were identified as suitable layouts for the website (Table 2). Furthermore, the structure of the leaflet and the titles of the poster and leaflet appeared to be very “catchy” and, therefore, were transferred to the first version of the website. Moreover, parts of the patient information folder and the one-pager, providing in-depth information on the isPO programme and isPO study, were perceived to be beneficial for the content of the website. This, for instance, included easy-to-understand explanations of all questionnaires for determining the patients’ needs and the presentation of the isPO programme and isPO study through the timeline. Therefore, both aspects were chosen to be utilized on the preliminary working version of the website. 

### 3.2. Outcomes from Step 3—Initial Exploration through Interviews

The interviewees (isPO-Oncoguides and isPO-Case Manager, Figure 2) also reflected on the existing four isPO PIM. Their reflections and statements were distinguished in three topics:

(1) Assessment of the existing information material: The structure of the flyer and the tree motif were perceived to be a positive and stimulating signal. The participants noted that the illustration of the chronological sequences of the questionnaires, as presented on the one-pager, was “too complex” and “overwhelming”, and thus hard to understand. Moreover, the content material of the preliminary working version of the isPO website was considered to be “too text-heavy”.
(2) Exploring a suitable patient-friendly design and structure of the website: Participants confirmed a positive association with the green colour scheme and the tree motif. In addition, they emphasized that we should consider a “good balance between text parts and illustrations”.
(3) Reflecting on the meaningful content of the website: All participants suggested that the stepped-care approach of isPO and the different professions involved in the care provision (isPO team) should be carefully elucidated. The interviewees emphasised the importance of offering comprehensive information on what psycho-oncological care is and entails, as “potentially many patients are unfamiliar with this care approach” in Germany and it, thus, may help diminish perceived stigmatisation around psychosocial distress among cancer patients.

### 3.3. Outcomes from Step 4—Creation of an Initial Working Version of the Website

Based on the results of the document analysis and the interviews (Figure 2, steps 2 and 3), a first working version of the website was created (Figure S1). The criteria for a user-friendly website according to Krug and Dubau [35] were applied during this process.

The structure of the first working version was very basic, consisting of the main page (home) and four topic pages: (1) isPO, (2) contact persons, (3) downloads, and (4) frequently asked questions (FAQ). In addition, a search function was added.

The isPO topic page (home) was subdivided into the following sections: (1) care programmes, (2) surveys, (3) participation, (4) data protection, and (5) information around the isPO project. The structure of the website contains a combined structure. There are elements of a tree structure, such as on the isPO topic page, as it leads to further sub-pages. In addition, the website also includes some aspects of a network structure, such as the integration of internal links (e.g., to the contact persons) and a search function.

### 3.4. Outcomes from Step 5—Optimisation Loop 1

In order to optimise the first version of the website, a focus group was conducted within a PAR cycle (Figure 2). The outcomes of the focus group and the interviews were divided into optimisation comments regarding the first working version of the *content, design and structure* of the website. The participants criticised the content of several text sections and the design of the integrated illustrations, as one figure on the website “is more of a procedural graphic for the project’s internal providers and rather not for the patients.” Suggestions for revision mainly concerned the structure of the website, which included the menu structure and the names of the page titles. It was said that “the term must clearly indicate what to be found behind a bottom.” Based on these very specific valuable suggestions, a comprehensive revision of the first working version of the website was realised. The optimisations are exemplified by the three loops (Table S2).

### 3.5. Outcomes from Step 5—Optimisation Loop 2

In the second optimisation loop (Figure 2), the interview participants were former cancer patients who are living outside the catchment area of isPO and, therefore, had no experiences with isPO and no information regarding the website beforehand. As in loop one, the overall statements of the interview participants were divided into the topics: content, design and structure. Contrary to loop one, the main focus lay on the content and design of the patient-friendly website. This included simplifying the wording of text elements and a more intuitive and comprehensive design of illustrations. The participants also offered clear suggestions as to how the functions of the website could become more patient-friendly. For instance, on the page describing the isPO care programme, one participant noted that not all people may recognise their levels of psychosocial distress correctly. It was said that “the direct jump to a psychotherapist can be so hard to some people that it may frighten them and consequently they would not join the care programme.” 

It was further noted that integrated text fields were still too long. Moreover, certain interactive elements of the website remained undiscovered, e.g., the greyed-out area should be clicked on in order to change the page of the questionnaires. Nevertheless, the overall layout of the website and the general ease of use were perceived to be very positive. The profusion of content was now described as appropriate, and the information was also seen to be relevant for family caregivers and the patients’ relatives. Based on this second PAR cycle, the second working version of the website was optimised again, taking the important issues in consideration. Finally, this second optimisation loop resulted in making the functions of the website clearly more user-friendly. 

### 3.6. Outcomes from Step 5—Optimisation Loop 3

In the third and final optimisation loop, the isPO-Oncoguides (knowing isPO itself very well as professionals and being former patients), were very content with this newly designed patient-friendly website and welcomed all the effort. However, certain terms and formulations still needed optimisation to be more understandable for the patients. For instance, instead of the term treatment, the German terms for “support or care” were noted to be more appropriate. Furthermore, in the section *care programme*, where the professions are explained, the isPO-Oncoguide was initially described as a “*former cancer patient*”. Two participants suggested a re-formulation, as a cancer patient may remain a patient even though its treatment is completed. The participants suggested to describe the isPO-Oncoguide, as “a person who has its own experience with cancer”. 

With regard to the design, the participants discovered minor errors in internal links and the formatting. Based on these results, a final revision of the website was realised. 

**Figure 3:**
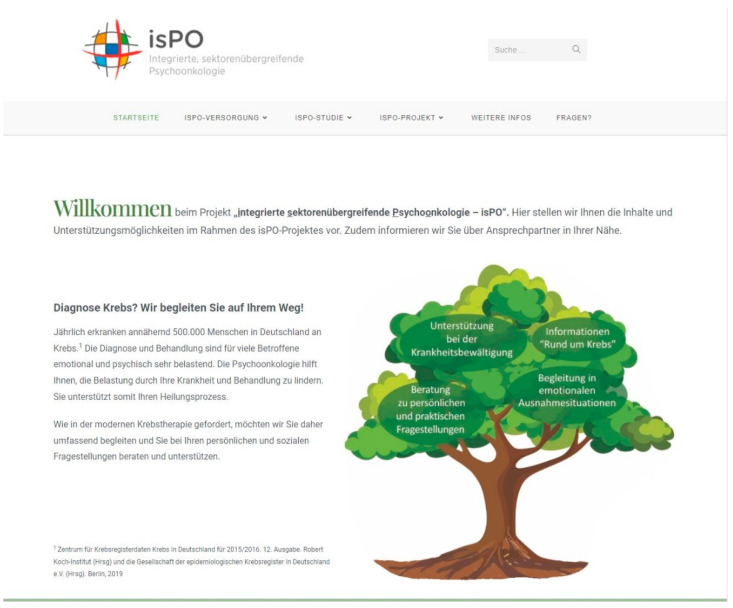
Final shape of the patient-friendly isPO website.

Figure 3 shows an excerpt of the final home page. This final version of the patient-friendly isPO website can be found using the following link: https://ispo.uni-koeln.de (accessed on 24 January 2022). 

### 3.7. Outcomes from Step 6—Plausibility Check

After the completion of the third optimisation loop, the plausibility of the website was assessed. Finally, 10 participants completed the UPIM-Check. Five of the participants were isPO-Oncoguides, and the other five were members of the HKSH-BV. The latter belong to different German cancer peer groups and did not have any affiliation or contact with isPO beforehand. The assessment of the website was conducted with the UPIM-Check. The quantitative evaluation of the checked fields was descriptive (Table 3).

Of all four quality criteria, Q3 *structural readability* and Q4 *graphical readability* were rated best on average, with 70% of respondents rating it as “very good”. Q1 *correctness and validity of the content* was rated lowest on average, with 44% rating it as “very good”. Finally, the free-text fields of the completed UPIM-Checks were evaluated qualitatively. There are several comments, particularly with regard to the quality criteria of Q1 *correctness and validity of content* and Q2 *readability of content*. In the case of the former, the main comment is that the website did not provide enough information to enable an outsider to understand the isPO project. In the aspect of contextual integration into the life situation, concrete patient examples were desired. 

## 4. Discussion

### 4.1. Principal Findings

This study demonstrates how a patient-friendly isPO website was designed “bottom-up” by applying the PHR approach based on the previously optimised isPO PIM [30]. Such a website was identified as the “final missing element“ to complete the isPO PIM [39]. It was perceived to be crucial for: (1) enhancing the patients’ understanding of the isPO care programme and the isPO study, (2) addressing and prioritising the end-users’ (newly diagnosed cancer patients) need for a digital platform (user-friendly accessibility of information), and (3) augmenting the programme and study recruitment processes. These demands were achieved by engaging with the retrospective end-users (patient representatives and cancer survivors), isPO service providers (isPO-Case Managers and isPO-Oncoguides), as well as experts from the cancer support network (KG-NRW) during the study. The various perspectives led to a comprehensive understanding of the real needs and perceptions regarding the required quality of a website. 

Our process was framed by the PHR approach [25] and constantly targeted high levels of participation [30]. The approach empowered the PHR research team to gain an enhanced understanding and fundamental insights into the requirements on how to design this patient-friendly isPO website. 

Step 3 of the research process paved the way for the creation of a first working version through the conduction of a collaborative assessment of the existing PIM. The initial exploration demonstrated the complexity of isPO and, hence, the importance to establish an easy-to-understand design (e.g., illustrations), content (e.g., text), and structure. In addition, the need for a sensitive approach to introduce isPO and elucidate on what psycho-oncological care entails became evident. On the basis of these insights, the first working version of the website was created (step 4). In step 5, three iterative optimisation loops using PAR were conducted to prioritize the main aspects (i.e., design, content, and structure), detect “teething problems” (i.e., difficult wording, function weaknesses), and assure a patient-friendly design. Finally, a plausibility check (step 6) illuminated the successful amendments of the design and structure of the website. 

### 4.2. Why a Patient-Friendly Website Is Needed

In isPO, a patient-friendly website was required by the PIM optimisation team to address the patients’ and their informal caregivers’ informational needs regarding the isPO care programme and the isPO study [23]. The importance of the provision of a digital PIM has been highlighted by several studies, since the internet has become an increasingly and successfully used source of health information for patients [14,15,16]. 

Although there was already a top-down developed isPO website available, it was addressed to service providers or researchers, rather than to the end-users’ needs and, thus, did not strengthen the acceptance of the programme. Another important aspect for the design of a patient-friendly website was that the other optimised PIM were analogue. Since the website would offer a different type of format (digital) and generate access to valuable information, it was seen to be indispensable. This perception is underpinned by evidence stating that patients have different preferences regarding learning and information formats [40,41]. We experienced that the acceptance of isPO could be improved, especially during the COVID-19 pandemic [42]. The notion of improved acceptance through an enhanced understanding of health information is supported by findings from Adnan, Warren and Suominen [43], which link an improved understanding of health information with the end-users’ empowerment to take a more active role in the decision-making process of the care they receive [43].

### 4.3. How to Design this Website

We promote the investment of sufficient time in designing, piloting, and optimising the website. It will lead to a mature product that really transmits the contained information (e.g., a new programme or a study) in a way that patients will welcome. Our product shows that PIM outlined in a website format have the potential to offer easy-to-access and understandable information. Moreover, information may incentivise patients to take action (e.g., to search for psycho-oncological support). In order to achieve these goals, we found that it was crucial to engage not only experts, but also potential programme users (e.g., patients or service providers) in its designing process. When initially inaugurating the website design process, different studies were analysed that turned out to be useful for this purpose. The study by Kildea et al. [6] offered interesting insights into their participative study approach. For instance, they included a patient among their co-researchers, which provides an innovative example of participation [6]. Although our study did not incorporate a patient as a co-researcher, the large variety of participants and high levels of participation during the data collection (PAR) assured a strong input with respect to the optimisation of the patient-friendly website. Additionally, the composition of participants that were familiar or unfamiliar with isPO allowed an authentic and reliable input into the optimisation of the main aspects (i.e., design, content, and structure) of the website. 

Further, the choice of a participatory study design in contrast to a top-down approach reflects on the notion to incorporate a multiplicity of perspectives that depict on the end-users’ needs. Insights by Unger [26] support this notion and reflect on the importance of a participatory study design in regard to acknowledging the patients’ experiences and needs [26]. The authors of this study consider that the research process was essentially driven in collaboration with the end-users, as they were truly invited to contribute with their unique perspective as “experts with experiences”. This is underpinned by Bergold and Thomas [44], who declare that research should not be conducted *about* or *for* the end-user, but it should rather be conducted *with* the end-user [44,45]. Due to our experiences in this study, we are certain that a high-quality patient-friendly website can only meet its required standards of success when its end-users (e.g., patients) are fully incorporated in the product’s design, which is also indicated by Beusekom et al. [46]. Additionally, besides strengthening the establishment of high-quality patient-friendly information material, it also demonstrates the researcher’s dedication to transform persistent health inequalities through the inclusion and emancipation of the end-users of the care programme [47]. 

### 4.4. Why the Plausibility Check was Necessary but Challenging

The plausibility check was perceived to be a final step to ensure the quality of the final product. Nevertheless, carrying out this step posed the researchers several challenges. The target group had to be contacted multiple times with detailed letters in order to induce them to participate in the plausibility check, and even then, the response rate remained low. In contrast to other countries, such as the United Kingdom, where clinical commissioning groups regularly involve patients to improve services [48], the researchers believe that patients in Germany are less experienced in participating in such participative processes. We believe that, in our case, the plausibility check might have led to an excessive demand and overburdening of the involved German cancer peer support group. In order to make care more patient-centred, the WHO has developed a guideline that emphasises the relevance of the patients’ voices [49]. This may help to combine the patients’ experiences with the knowledge of the experts, and to help patients in making their voices feel counted and the research outcomes more valuable. However, our study contributed to the empowerment of the peer groups, as they received the positive effects when they were involved in the research. 

### 4.5. Methodological Issues (PHR and PAR)

Participation is processual, and achieving high levels of participation requires both the PHR team and the engaged participants to become familiar with participatory research approaches that establish a degree of confidence so that the voices of “experts with own experiences” really count [50]. Those experts who were already used to PHR (i.e., isPO-Oncoguides) felt more confident in articulating their ideas and being creative. The PHR “newcomers” from the two peer support groups required more time and motivational support to feel confident with participation at levels four and five. Maybe further training and more information about this approach might help them beforehand, which is also emphasized by Unger [51]. However, due to the COVID-19 pandemic challenges and time constrains (Master’s degree qualification), it was difficult to even find suitable participants. As we could not apply the golden standard (face-to-face interactions), we were flexible with the way of collecting data (e.g., enable ZOOM meetings instead of face-to-face meetings) and would propose being flexible to other researchers. Further, we promote PHR training for professionals that are aiming to work with peer support groups in order to have the necessary skills and experience with this approach. This includes not only the knowledge of using different instruments, but also the skills to share power, manage conflict, or take on new roles [24,50]. 

### 4.6. Strength and Limitations

Our website design process was conducted “bottom-up” with the PHR approach, including three PAR loops. We empowered participants to contribute with high participation levels (e.g., co-creative learning), resulting in a website that really meets the requirements of the patients. Therefore, opposed to a “top-down” approach, PHR requires more resources (e.g., time, funding, or training). Applying PHR requires positive democratic experiences from all participants, as well as an open mindset and the understanding of the PHR approach (e.g., iterative decision-making process). Especially in the beginning, it might be characterised by uncertainty, as it remains unclear how many loops might be necessary or how many participants might be required, as outcomes depend on the participants’ experiences and abilities. 

A strength of our website is that it was created with a comprehensive multi-perspective understanding. We incorporated knowledge from: (1) “experts with own experiences” from two different peer groups and isPO-Oncoguides, (2) professional isPO service providers, (3) experts in cancer support (KG-NRW), and (4) research experts with PHR experiences (with IMVR as a critical friend). 

Finally, our website is very context-specific. It was designed to the specific needs of newly detected German adult cancer patients that are interested in understanding and attending psycho-oncological care and this specific isPO programme. Hence, direct transfer to other programmes might be challenging (e.g., language, specific approach, paediatric oncology). Still, we are certain that our website may serve as a template. 

## 5. Conclusions

The patient-friendly website completes the optimised PIM of the isPO project as the fifth and final component. With this digital PIM, patients and other interested parties have easier access to high-quality information about the isPO care programme and isPO study. With the innovative utilisation of the PHR approach, this study sets an example on how to design a patient-friendly website that includes isPO’s end-users with high participation levels. PHR enabled an in-depth understanding and co-creation of knowledge together with retrospective end-users (isPO-Oncoguides). An iterative design process involving potential or respective end-users (cancer patients) will increase the chance of gaining a mature and “really” patient-friendly product. With the involvement of patient representatives, isPO-Oncoguides, an isPO-Case Manager, and health professionals, a rich inclusion of various perspectives was given. This study highly recommends the design, piloting, and optimisation of a website for patients with early and strong end-user engagement by applying the PHR approach. 

## Figures and Tables

**Table 1:** Characteristics of the study participants.

Participant and Background	Sex (F/M)	Type (I/FG)	Setting/Affiliation
*isPO-Oncoguide* Former cancer patient	F	Interview	Johanna Etienne Hospital Neuss
*isPO-Oncoguide* Former cancer patient	M	Interview	University Hospital of Cologne
*isPO-Oncoguide* Former cancer patient	F	Interview	Maria Hilf Hospital Mönchengladbach
*isPO-Case Manager* The patient’s contact person	F	Interview	University Hospital of Cologne
*Experts* In the cancer support sector	F (*n* = 3)	Focus Group	Members of KG NRW
*Potential end-users* Former cancer patient	F	Interview	Member of the House of Cancer Self-Help
*Potential end-users* Former cancer patient	F	Interview	Member of the House of Cancer Self-Help
*Potential end-users* Former cancer patient	M	Interview	Member of the District Association of Laryngectomists Aachen e.V.
*Potential end-users* Former cancer patient	F	Interview	Member of the District Association of Laryngectomists Aachen e.V.
*Potential end-users* Former cancer patient	M	Interview	Member of the District Association of Laryngectomists Aachen e.V.
*isPO-Oncoguides* Former cancer patients	F (*n* = 5) M (*n* = 2)	Focus Group	University Hospital of Cologne, Johanna Etienne Hospital Neuss, Maria Hilf Hospital Mönchengladbach, GFO Troisdorf Hospital

**Table 2:** Outcomes of the document analysis: description of the isPO PIM and its website suitability.

isPO PIM (Optimised from 03/2021)	Utilisation in isPO	Content	Design Features	Suitable Element for Utilisation on the Patient-Friendly Website
Poster	For all patients (e.g., in the waiting room)	Mention of rough key data; the description in key points; call to address in case of interest	Tree motif; green colour scheme; isPO logo	Tree motif Slogan “Cancer diagnosis? We accompany you on your way!” isPO logo
Leaflet	For potential isPO patients and interested parties (e.g., distributed on oncological units)	Explanation of what isPO is, what the care looks like, what patients receive in the care; specific contacts	Tree motif; green colour design	Title of the flyer “Psycho-oncological support, counselling, treatment for patients with an initial diagnosis of cancer” The structure of the flyer (What is it? What does care look like? Who can participate? Contact persons) Flyer could be linked to the website as a document
Patient information folder	The potential isPO patients receive the patient map during the educational talk (handover by the isPO-Case Manager)	Information on the isPO care programme and the isPO study	Four-page information map with isPO logo	Explanation of the questionnaires to determine the individual need for psycho-oncological support Very comprehensible information about the programme and study
“One-Pager” (study information overview)	isPO patients receive the one-pager when enrolling in the study (handover by the isPO-Case Manager)	Description of the isPO study	Coloured timeline of the isPO programme and isPO study	Timeline presentation with different colours for programme and study Present the meaning of the evaluation study topic data protection

**Table 3:** Descriptive statistical evaluation of the UPIM-Checks.

	Correctness & Validity of Content - Does the Content Seem to Be Correct? Does the Information Appear to Be Valid?										
	Q1	Q1.1	Q1.2	Q1.3	Q1.4	Q1.5	Q1.6	Q1.7	Q1.8	Q1.9	
**Very Good**	44.4%	60.0%	50.0%	80.0%	30.0%	70.0%	40.0%	40.0%	20.0%	10.0%	
**Sufficient**	24.4%	20.0%	20.0%	0.0%	40.0%	0.0%	20.0%	30.0%	40.0%	50.0%	
**Unsatisfactory**	17.8%	0.0%	20.0%	10.0%	10.0%	20.0%	30.0%	10.0%	30.0%	30.0%	
**NI**	13.3%	20.0%	10.0%	10.0%	20.0%	10.0%	10.0%	20.0%	10.0%	10.0%	
	**Readability of Content - Is the Content Easy to Read?**										
	**Q2**	**Q2.1**	**Q2.2**	**Q2.3**	**Q2.4**	**Q2.5**	**Q2.6**	**Q2.7**	**Q2.8**		
**Very Good**	50.0%	50.0%	60.0%	70.0%	60.0%	30.0%	60.0%	50.0%	20.0%		
**Sufficient**	25.0%	30.0%	10.0%	10.0%	20.0%	50.0%	20.0%	40.0%	20.0%		
**Unsatisfactory**	10.0%	20.0%	20.0%	10.0%	10.0%	0.0%	10.0%	0.0%	10.0%		
**NI**	15.0%	0.0%	10.0%	10.0%	10.0%	20.0%	10.0%	10.0%	50.0%		
	**Structural Readability - Is the Structure of the Information Appropriate for the Target Group?**										
	**Q3**	**Q3.1**	**Q3.2**	**Q3.3**	**Q3.4**						
**Very Good**	70.0%	80.0%	80.0%	80.0%	40.0%						
**Sufficient**	17.5%	10.0%	10.0%	10.0%	40.0%						
**Unsatisfactory**	2.5%	0.0%	0.0%	0.0%	10.0%						
**NI**	10.0%	10.0%	10.0%	10.0%	10.0%						
	**Graphical Readability - Is the Layout Addressing the Needs of the Target Group?**										
	**Q4**	**Q4.1**	**Q4.2**	**Q4.3**	**Q4.4**	**Q4.5**	**Q4.6**	**Q4.7**	**Q4.8**	**Q4.9**	**Q4.10**
**Very Good**	70.0%	50.0%	70.0%	70.0%	70.0%	70.0%	60.0%	70.0%	80.0%	90.0%	70.0%
**Sufficient**	12.0%	20.0%	0.0%	10.0%	10.0%	10.0%	20.0%	20.0%	10.0%	0.0%	20.0%
**Unsatisfactory**	7.0%	30.0%	10.0%	10.0%	10.0%	10.0%	0.0%	0.0%	0.0%	0.0%	0.0%
**NI**	11.0%	0.0%	20.0%	10.0%	10.0%	10.0%	20.0%	10.0%	10.0%	10.0%	10.0%

## Data Availability

The data presented in this study are available on reasonable request from the corresponding author. The data are not publicly available due to ethical and legal restrictions (participants of this study did not agree for their data to be shared publicly).

## Supplementary Materials

The following supporting information can be downloaded at: https://www.mdpi.com/article/10.3390/ijerph19041969/s1, Table S1: The UPIM-Check checklist [1,8,9,10,24,52]; Table S2: The main topics of the revision of the optimization loops; Figure S1: the first working version of the website.

## References

[1] Sänger S., Lang B., Klemperer D., Thomeczek C., Dierks M.-L. (2006). Manual Patienteninformation: Empfehlungen zur Erstellung Evidenzbasierter Patienteninformationen.

[2] Coulter A. (1998). Evidence based patient information. Is important, so there needs to be a national strategy to ensure it. BMJ.

[3] Ernstmann N., Sautermeister J., Halbach S., Haring R. (2018). Gesundheitskompetenz. Gesundheitswissenschaften.

[4] Banasiak N.C., Meadows-Oliver M. (2017). Evaluating asthma websites using the Brief DISCERN instrument. J. Asthma Allergy.

[5] Tait A.R., Voepel-Lewis T., Levine R. (2015). Using digital multimedia to improve parents’ and children’s understanding of clinical trials. Arch. Dis. Child..

[6] Kildea J., Battista J., Cabral B., Hendren L., Herrera D., Hijal T., Joseph A. (2019). Design and Development of a Person-Centered Patient Portal Using Participatory Stakeholder Co-Design. J. Med. Internet Res..

[7] Roberto A., Colombo C., Candiani G., Satolli R., Giordano L., Jaramillo L., Castagno R., Mantellini P., Falini P., Carnesciali E. (2020). A dynamic web-based decision aid to improve informed choice in organised breast cancer screening. A pragmatic randomised trial in Italy. Br. J. Cancer.

[8] Herm K., Linden M. (2013). Qualitätssicherung von schriftlichen Patienteninformationen. Psychother. Psych. Med..

[9] Charnock D., Shepperd S., Needham G., Gann R. (1999). DISCERN: An instrument for judging the quality of written consumer health information on treatment choices. J. Epidemiol. Community Health.

[10] Shoemaker S.J., Wolf M.S., Brach C. (2014). Development of the Patient Education Materials Assessment Tool (PEMAT): A new measure of understandability and actionability for print and audiovisual patient information. Patient Educ. Couns..

[11] Köpke S., Berger B., Steckelberg A., Meyer G. (2005). In Deutschland gebräuchliche Bewertungsinstrumente für Patienteninformationen - eine kritische Analyse. Z. Ärztl. Fortbild. Qual. Gesundh. Wes..

[12] Salm S., Mollenhauer J., Hornbach C., Cecon N., Dresen A., Houwaart S., Arning A., Göttel A., Schwickerath K., Pfaff H. (2021). Participatory Development and Preliminary Psychometric Properties of the User-Friendly Patient Information Material Checklist (UPIM-Check). Int. J. Environ. Res. Public Health.

[13] Salm S. (2020). Optimizing the patient information material for cancer patients using the participatory health research approach. Eur. J. Public Health.

[14] Pew Research Center Health Topics. https://www.pewresearch.org/internet/2011/02/01/health-topics-4/.

[15] Amante D.J., Hogan T.P., Pagoto S.L., English T.M., Lapane K.L. (2015). Access to care and use of the Internet to search for health information: Results from the US National Health Interview Survey. J. Med. Internet Res..

[16] Reifegerste D., Bachl M., Baumann E. (2017). Surrogate health information seeking in Europe: Influence of source type and social network variables. Int. J. Med. Inform..

[17] Snyder C.F., Wu A.W., Miller R.S., Jensen R.E., Bantug E.T., Wolff A.C. (2011). The role of informatics in promoting patient-centered care. Cancer J..

[18] Soobrah R., Clark S.K. (2012). Your patient information website: How good is it?. Colorectal Dis..

[19] Risk A., Petersen C. (2002). Health information on the internet: Quality issues and international initiatives. JAMA.

[20] Refai M., Andolfi M., Gentili P., Pelusi G., Manzotti F., Sabbatini A. (2018). Enhanced recovery after thoracic surgery: Patient information and care-plans. J. Thorac. Dis..

[21] Jenniches I., Lemmen C., Cwik J.C., Kusch M., Labouvie H., Scholten N., Gerlach A., Stock S., Samel C., Hagemeier A. (2020). Evaluation of a complex integrated, cross-sectoral psycho-oncological care program (isPO): A mixed-methods study protocol. BMJ Open.

[22] Kusch M., Labouvie H., Schiewer V., Talalaev N., Cwik J.C., Bussmann S., Vaganian L., Gerlach A., Dresen A., Cecon N. (2020). Integrated, cross-sectoral psycho-oncology (isPO): A new form of care for newly diagnosed cancer patients in Germany. Manuscr. Submitt. Publ..

[23] Salm S., Houwaart S. (2020). 32.K. Skills building seminar: Using participatory health research to optimise psycho-oncological patient information material. Eur. J. Public Health.

[24] Krieger T., Salm S., Dresen A., Arning A., Schwickerath K., Göttel A., Houwaart S., Pfaff H., Cecon N. (2022). Patient’s perspective matters: Optimising patient information material for a new psycho-oncological care programme using a Participatory Health Research Approach in Germany. Int. J. Environ. Res. Public Health.

[25] Wright M.T. (2013). Was ist Partizipative Gesundheitsforschung?. Prävention Und Gesundh..

[26] von Unger H. (2014). Partizipative Forschung: Einführung in die Forschungspraxis.

[27] International Collaboration for Participatory Health Research (2013). Position Paper 1: What is Participatory Health Research? Mai 2013.

[28] Arnstein S.R. (1969). A Ladder Of Citizen Participation. J. Am. Inst. Plan..

[29] Pretty J.N. (1995). Participatory learning for sustainable agriculture. World Dev..

[30] Cornwall A. (1996). Part IV Participatory Research Methods: First Steps in a Participatory Process: Towards Participatory Practice: Participatory Rural Appraisal (PRA) and the Participatory Process. ParticipatoryResearch in Health: Issues and Experiences.

[31] Curry L., Nunez-Smith M. (2015). Mixed Methods in Health Sciences Research: A Practical Primer.

[32] Fetterman D. (2009). Empowerment evaluation at the Stanford University School of Medicine: Using a critical friend to improve the clerkship experience. Ens. Avaliação E Políticas Públicas Em Educ..

[33] Baum F., MacDougall C., Smith D. (2006). Participatory action research. J. Epidemiol. Community Health.

[34] Eisenmenger R. (2020). WordPress 5: Das Umfassende Handbuch.

[35] Krug S., Dubau J. (2006). Don’t make Me Think!: Web Usability—Das Intuitive Web.

[36] Krieger T., Salm S., Mollenhauer J., Cecon N., Dresen A., Houwaart S., Schwickerath K., Göttel A., Arning A. (2021). UPIM-Check: User-friendly Patient Information Material Checklist. https://www.imvr.de/wp-content/uploads/UPIM-Check_English.pdf.

[37] Braun V., Clarke V. (2006). Using thematic analysis in psychology. Qual. Res. Psychol..

[38] Mayring P. (1999). Einführung in die Qualitative Sozialforschung: Eine Anleitung zu Qualitativem Denken.

[39] Krieger T., Salm S., Cecon N., Pfaff H., Dresen A. (2021). Ergebnisbericht der Zweiten Externen Formativen Evaluation des Projekts isPO: Forschungsbericht 03-2021.

[40] Ream E., Blows E., Scanlon K., Richardson A. (2009). An investigation of the quality of breast cancer information provided on the internet by voluntary organisations in Great Britain. Patient Educ. Couns..

[41] Vogel B.A., Bengel J., Helmes A.W. (2008). Information and decision making: Patients’ needs and experiences in the course of breast cancer treatment. Patient Educ. Couns..

[42] Krieger T., Salm S., Cecon N., Pfaff H., Dresen A. (2021). Vorläufige Summative Evaluation des Projekts isPO: Forschungsbericht 10-2021.

[43] Adnan M., Warren J., Suominen H., Grando M., Rozenblum R., Bates D. (2015). 10 Patient empowerment via technologies for patient-friendly personalized language. Information Technology for Patient Empowerment in Healthcare.

[44] Bergold J., Thomas S., Mey G., Mruck K. (2020). Partizipative Forschung. Handbuch Qualitative Forschung in der Psychologie.

[45] Cornwall A. (2008). Unpacking ’Participation’ Models, meanings and practices. Community Dev. J..

[46] van Beusekom M.M., Kerkhoven A.H., Bos M.J.W., Guchelaar H.-J., van den Broek J.M. (2018). The extent and effects of patient involvement in pictogram design for written drug information: A short systematic review. Drug Discov. Today.

[47] Torre M.E., Ayala J. (2009). Envisioning Participatory Action Research Entremundos. Fem. Psychol..

[48] Gillam S., Newbould J. (2016). Patient participation groups in general practice: What are they for, where are they going?. BMJ.

[49] Castro E.M., van Regenmortel T., Vanhaecht K., Sermeus W., van Hecke A. (2016). Patient empowerment, patient participation and patient-centeredness in hospital care: A concept analysis based on a literature review. Patient Educ. Couns..

[50] Bergold J., Thomas S. (2012). Participatory Research Methods: A Methodological Approach in Motion. Hist. Soc. Res. Hist. Soz..

[51] von Unger H. (2012). Partizipative Gesundheitsforschung: Wer partizipiert woran?. Forum Qual. Soz. Forum: Qual. Soc. Res..

[52] Zhang Y., Sun Y., Xie B. (2015). Quality of health information for consumers on the web: A systematic review of indicators, criteria, tools, and evaluation results. J. Assoc. Inf. Sci. Technol..

