# Supplementary material for: Designing a Patient-Friendly Website for Newly Diagnosed Cancer Patients with the Participatory Health Research Approach
Source: Int J Environ Res Public Health. 2022 Feb 10;19(4):1969. doi: 10.3390/ijerph19041969 (PMC8872440; doi:10.3390/ijerph19041969)
Supplement: Supplementary file 1 [file ijerph-19-01969-s001.zip › ijerph-1545346-supplementary.pdf]

Supplementary Materials

Table S1 UPIM-Check (User-friendly Patient Information Material Checklist – English Version)[24]

Instrument to assess and optimise the quality of patient information material (PIM)

**Document name** (e.g. short informational flyer):					
**Reviewer:** Person affected (e.g. self-help) Provider (e.g. case manager, psychotherapist)		Expert (e.g. academic researcher, project staff)			
**Where and how the patient comes into contact with PIM:**					
**Quality criteria**		**very good**	**suffi-cient**	**unsatis-factory**	**Suggestions for improvement**
**Q1: Correctness & validity of content – Does the content seem to be correct? Does the information appear to be valid?**					
**Q1.1**	**Up-to-date & technically correct**<br>(references, expertise of the authors, date)<br>*Does the information appear to be up-to-date?Is the information correctly cited?*	☐	☐	☐	
**Q1.2**	**Transparency**<br>(author of the PIM; contact person, contact & logo)<br>*How clear is the information?*	☐	☐	☐	
**Q1.3**	**Information is relevant for the target group**<br>(social evidence)<br>*Is the information relevant for the target group?*	☐	☐	☐	
**Q1.4**	**Contextual integration into patient's situation**<br>(experience, emotions, burden)<br>*How does the information fit the patient's situation?*	☐	☐	☐	
**Q1.5**	**Focus**<br>(only 1-2 aspects, e.g. cancer and holisticsupport)<br>*Is the focus of the content clear and easy toidentify?*	☐	☐	☐	

© 2020, Theresia Krieger, Sandra Salm, Judith Mollenhauer, Natalia Cecon, Antje Dresen, Stefanie Houwaart, Kathrin Schwickerath, Andrea Göttel, Anna Arning
Developed in a participatory process by: University of Cologne, House of the Cancer Patient Support Associations of Germany, Cancer Society North Rhine-Westphalia.

**Q1.6**	**Adequate presentation**<br>(benefits, risks and impacts are adequately presented for decision making)<br>*Is the information presented in a way that aninformed decision can be made?*	☐	☐	☐	

© 2020, Theresia Krieger, Sandra Salm, Judith Mollenhauer, Natalia Cecon, Antje Dresen, Stefanie Houwaart, Kathrin Schwickerath, Andrea Göttel, Anna Arning
Developed in a participatory process by: University of Cologne, House of the Cancer Patient Support Associations of Germany, Cancer Society North Rhine-Westphalia.

Quality criteria		very good	suffi-cient	unsatis-factory	Suggestions for improvement
**Q1: Correctness & validity of content – Does the content seem to be correct? Does the information appear to be valid?**					
Q1.7	**Motivation & increase of self-efficacy** (the patient is interested in the offer (e.g. information, programme) because...) *Does the information increase the readersmotivation to act?*	☐	☐	☐	
Q1.8	**Recommendation for action** (turning information into activity) *Does the information recommend clear steps foraction?*	☐	☐	☐	
Q1.9	**Further literature / points of contact** (further information is accessible, in other words, no "dead" links; contact person if information is not available) *Does the information provide further sources for reading? Is a point of contact mentioned?*	☐	☐	☐	

Q2: Readability of content – Is the content easy to read?					
Q2.1	**Aim of the PIM and target group is identifiable** *Are the aim and the target group of the PIM clear?*	☐	☐	☐	
Q2.2	**Clarity of content** (short and concise, inspiring content e.g. quotations) *Is the content clear for the target group?*	☐	☐	☐	
Q2.3	**Simple, clear language** *Is the PIM written in a simple and clear way?*	☐	☐	☐	
Q2.4	**Neutral language** (non-directive) *Is the PIM presented in an open-minded, not manipulating way?*	☐	☐	☐	

© 2020, Theresia Krieger, Sandra Salm, Judith Mollenhauer, Natalia Cecon, Antje Dresen, Stefanie Houwaart, Kathrin Schwickerath, Andrea Göttel, Anna Arning
Developed in a participatory process by: University of Cologne, House of the Cancer Patient Support Associations of Germany, Cancer Society North Rhine-Westphalia.

Quality criteria		very good	suffi-cient	unsatis-factory	Suggestions for improvement
**Q2: Readability of content – Is the content easy to read for the target group?**					
**Q2.5**	**Target group-specific language**<br>(age, education, health literacy)<br>*Does the language fit the target group?*	☐	☐	☐	
**Q2.6**	**Use of numbers**<br>(numbers used are clear and easy to understand; no calculations)<br>*Are the numbers used easy to understand?*	☐	☐	☐	
**Q2.7**	**Language that can be understood without prior medical knowledge,**<br>otherwise medical terms must be defined<br>*Is the information understandable without priormedical knowledge?*	☐	☐	☐	
**Q2.8**	**Use of empowering words**<br>*Does the information use words that strengthenthe target group?*	☐	☐	☐	

**Q3: Structural readability – Is the structure of the information appropriate for the target group?**					
**Q3.1**	**Sentence length**<br>*Is the length of the sentences appropriate for thetarget group?*	☐	☐	☐	
**Q3.2**	**Sentence difficulty/complexity**<br>*Is the sentence structure appropriate for the target group?*	☐	☐	☐	
**Q3.3**	**Word length**<br>*Is the length of the words appropriate for the target group?*	☐	☐	☐	
**Q3.4**	**Word difficulty**<br>*Are the words utilised appropriate?*	☐	☐	☐	

© 2020, Theresia Krieger, Sandra Salm, Judith Mollenhauer, Natalia Cecon, Antje Dresen, Stefanie Houwaart, Kathrin Schwickerath, Andrea Göttel, Anna Arning
Developed in a participatory process by: University of Cologne, House of the Cancer Patient Support Associations of Germany, Cancer Society North Rhine-Westphalia.

Quality criteria		very good	suffi-cient	unsatis-factory	Suggestions for improvement
**Q4: Graphical readability – Is the layout addressing the needs of the target group?**					
**Q4.1**	**Layout / overall visual appearance**<br>*Is the layout of the text and the overall look ofthe material satisfactory?*	☐	☐	☐	
**Q4.2**	**Eye-catching**<br>(catchy title, picture)<br>*Does the material include eye-catching elements?*	☐	☐	☐	
**Q4.3**	**Appropriate overall text length**<br>*Is the length of the text appropriate for the target group?*	☐	☐	☐	
**Q4.4**	**Structure and context**<br>(e.g. information is condensed into short sections; sections have informative headings; information is arranged in a meaningful and logical order; summary)<br>*Is the PIM structured in a logical and meaningful order?*	☐	☐	☐	
**Q4.5**	**Illustrations**<br>(pictures, graphics)<br>*Are the pictures and graphics used concise and understandable?*	☐	☐	☐	
**Q4.6**	**Coloured headings and highlighting of key points**<br>*Are coloured headings and highlighting of keypoints used meaningfully?*	☐	☐	☐	
**Q4.7**	**Font size**<br>(min. size 12)<br>*Is the font size appropriate?*	☐	☐	☐	

© 2020, Theresia Krieger, Sandra Salm, Judith Mollenhauer, Natalia Cecon, Antje Dresen, Stefanie Houwaart, Kathrin Schwickerath, Andrea Göttel, Anna Arning
Developed in a participatory process by: University of Cologne, House of the Cancer Patient Support Associations of Germany, Cancer Society North Rhine-Westphalia.

**Q4.8**	**Font colour**<br>(e.g. contrast with background; web links can be distinguished from "normal" text)<br>*Are the font colours meaningful applied?*	☐	☐	☐	

© 2020, Theresia Krieger, Sandra Salm, Judith Mollenhauer, Natalia Cecon, Antje Dresen, Stefanie Houwaart, Kathrin Schwickerath, Andrea Göttel, Anna Arning
Developed in a participatory process by: University of Cologne, House of the Cancer Patient Support Associations of Germany, Cancer Society North Rhine-Westphalia.

Quality criteria		very good	suffi-cient	unsatis-factory	Suggestions for improvement
**Q4: Graphical readability – Is the layout addressing the needs of the target group?**					
**Q4.9**	**Font type**<br>(plain font, e.g. Arial)<br>*Is the font type easy to read?*	☐	☐	☐	
**Q4.10**	**Corporate design**<br>(recognisable, e.g. logo)<br>*Is a corporate design identifiable?*	☐	☐	☐	

Condensed instrument based on the work of Charnock et al. (1999); Herm & Linden (2013); Sänger et al. (2006); Shoemaker et al. (2014); Zhang et al. (2015). [1,8,9,10,52]

© 2020, Theresia Krieger, Sandra Salm, Judith Mollenhauer, Natalia Cecon, Antje Dresen, Stefanie Houwaart, Kathrin Schwickerath, Andrea Göttel, Anna Arning
Developed in a participatory process by: University of Cologne, House of the Cancer Patient Support Associations of Germany, Cancer Society North Rhine-Westphalia.

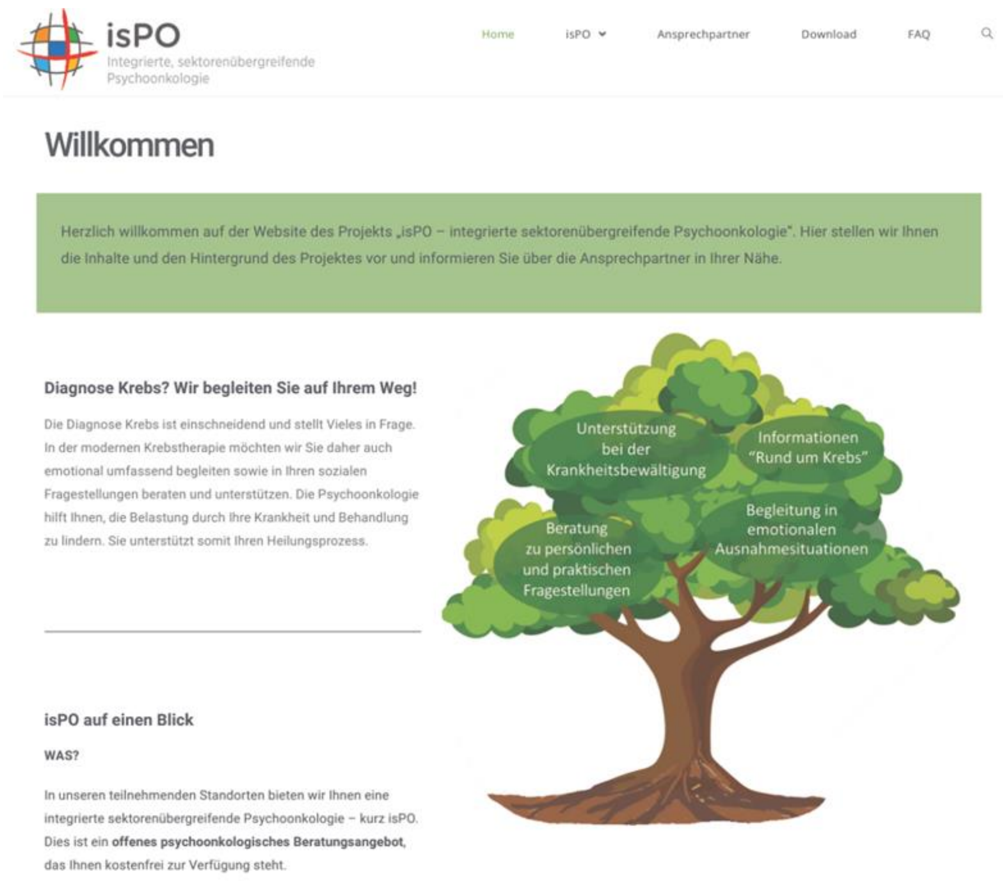

Figure S1. the first working version of the website

Table S2. The main topics of the revision of the optimisation loops

1st Optimisation loop	2nd Optimisation loop	3rd Optimisation loop
Clear and easy to understand differentiation of: (1) the isPO project, (2) stepped-care programme isPO, and (3) isPO study	Simplification of some wording and shortages of sentences (e.g. the incentive to click to a further subtopic-page is higher when there are shorter sentences used)	Amendment of individual terms for improved comprehensibility (e.g. instead of the term treatment the terms support or care should be used)
Major revision (simplification) of the menu structure and the page titles	Further minor optimisation of illustrations in order to make them intuitive understandable (e.g. colour, complexity reduction)	Revision of individual formulations<br>(e.g. the isPO-Oncoguide does not fully comprise a former cancer patient. The suggestion for "a person who has experience with the disease" was provided.)
Revision of formulations and texts in terms of content to make it user-friendly	Augmentation of the website's functions to make it more user-friendly (e.g. FAQ changed into the German word "Fragen")	Revision of incorrect internal links<br>(e.g. to the isPO networks)
Fundamental optimisation and or redesign illustrations/figures		Fixing errors in the formatting<br>(e.g. size of sub headings)
